# Species-specific shifts in centromere sequence composition are coincident with breakpoint reuse in karyotypically divergent lineages

**DOI:** 10.1186/gb-2007-8-8-r170

**Published:** 2007-08-20

**Authors:** Kira V Bulazel, Gianni C Ferreri, Mark DB Eldridge, Rachel J O'Neill

**Affiliations:** 1 Department of Molecular and Cell Biology, Mansfield Rd, University of Connecticut, Storrs, CT 06269, USA; 2 Department of Biological Sciences, Macquarie University, NSW 2109, Australia; 3 Molecular Biology, Australian Museum, College St, Sydney, NSW 2010, Australia

## Abstract

**Background:**

It has been hypothesized that rapid divergence in centromere sequences accompanies rapid karyotypic change during speciation. However, the reuse of breakpoints coincident with centromeres in the evolution of divergent karyotypes poses a potential paradox. In distantly related species where the same centromere breakpoints are used in the independent derivation of karyotypes, centromere-specific sequences may undergo convergent evolution rather than rapid sequence divergence. To determine whether centromere sequence composition follows the phylogenetic history of species evolution or patterns of convergent breakpoint reuse through chromosome evolution, we examined the phylogenetic trajectory of centromere sequences within a group of karyotypically diverse mammals, macropodine marsupials (wallabies, wallaroos and kangaroos).

**Results:**

The evolution of three classes of centromere sequences across nine species within the genus *Macropus *(including *Wallabia*) were compared with the phylogenetic history of a mitochondrial gene, *Cytochrome b *(*Cyt b*), a nuclear gene, *selenocysteine tRNA *(*TRSP*), and the chromosomal histories of the syntenic blocks that define the different karyotype arrangements. Convergent contraction or expansion of predominant satellites is found to accompany specific karyotype rearrangements. The phylogenetic history of these centromere sequences includes the convergence of centromere composition in divergent species through convergent breakpoint reuse between syntenic blocks.

**Conclusion:**

These data support the 'library hypothesis' of centromere evolution within this genus as each species possesses all three satellites yet each species has experienced differential expansion and contraction of individual classes. Thus, we have identified a correlation between the evolution of centromere satellite sequences, the reuse of syntenic breakpoints, and karyotype convergence in the context of a gene-based phylogeny.

## Abstract 2

The evolution of three classes of centromere sequences across nine species of macropodine marsupials were compared with that of other genes, showing that each species has experienced differential expansion and contraction of individual classes.

## Background

The centromere paradox posits that the DNA at centromeres is conserved for function, but not sequence [1]. Within the murine and primate lineages, centromeric DNA sequences are species specific and different chromosomes within a species sometimes contain divergent centromeric DNA sequences [2]. In stark contrast, the gross structure of the centromere and the associated kinetochore proteins are conserved across eukaryotes [3,4]. Such functional conservation in the apparent absence of sequence conservation, combined with the identification of functional centromeres at non-centromere locations (that is, neocentromeres), has led to the hypothesis that centromeres are largely determined by epigenetic modifications, such as histone variants [5,6] (reviewed in [7]). In humans, it has been suggested that segmental duplication events around the centromere ultimately lead to the high degree of variability within centric sequences [8,9]. However, studying the evolution of human centromere sequences in the context of karyotypic change has been difficult because the family of great apes has experienced little gross chromosome change between species [10].

Several marsupial families have experienced extensive karyotypic change, deriving from the rearrangement of a basic complement of 19 chromosome blocks through centric shifts (centromere repositioning), fissions, fusions and translocations [11-13]. Extant marsupial karyotypes exhibit a bimodal distribution between 2n = 14 and 2n = 22 [14,15]. While the 2n = 14 karyotype is homologous in several extant lineages, the 2n = 22 karyotype is highly divergent, suggesting independent derivation through breakpoint reuse. Rens *et al*. [12] traced the history of the rearrangements of these 19 syntenic blocks across several marsupial families, demonstrating frequent convergent breakpoint reuse within marsupials at breaks of synteny between these chromosome segments [11,12].

The recent radiation of karyotypically diverse species within the marsupial subfamily Macropodinae (kangaroos, wallaroos and wallabies) [11] affords the opportunity to study centromere evolution in the context of karyotypic change within a relatively short evolutionary time frame. Across the approximately 58 macropodine species diploid numbers range from 2n = 10(XX)/11(XY_1_Y_2_) (*Wallabia bicolor*) to 2n = 24 (*Lagostrophus fasciatus*), all derived through different suites of centric fusions (Robertsonian translocations), centric shifts (centromere repositioning) and pericentric inversions [16,17]. Within the Macropodinae, the genus *Macropus *(14 species including *W. bicolor*) has undergone a recent (4-11 million years ago) [18-20] and rapid karyotypic radiation. However, phylogenetic studies within this genus, relying on DNA-DNA hybridization [21], chromosome evolution based on G-banding studies [13] and serology-based studies [22,23] have failed to provide well-supported concordant phylogenies for species within this genus.

Confounding efforts to reconstruct phylogenetic relationships based on chromosome evolution is the observation that several species within *Macropus *have experienced breakpoint reuse between syntenic blocks, each at active centromere locations, in the derivation of novel karyotypes (reviewed in [11]). For example, the karyotype of the model species *Macropus eugenii *(tammar wallaby) is derived from the ancestral 2n = 22 through a series of fusions and translocations resulting in a reduction in chromosome number to 2n = 16. In fact, three different 2n = 16 karyotypes are seen within *Macropus*, each resulting from different fusions and translocations at the centromeres of the same syntenic blocks. The reuse of the breaks of synteny within this genus occurs exclusively at centromeric sites, allowing commensurate tracking of syntenic boundaries and centromeric sequences.

We hypothesize that the reuse of breaks of synteny involving centromeric sequences, active or inactive, leads not to an increase in the variability of involved DNA sequences, but instead leads to their conservation. The 'library hypothesis' posits that a suite of satellite sequences may be shared between closely related species [24]. Different satellite families may experience different evolutionary processes, such as concerted evolution, intrachromosomal sequence conversion and unequal crossing over [25]. Species specific 'turnover' of these sequences may occur when a satellite family becomes selected as a major centromere satellite capable of attracting centromere proteins, and is thus functional with respect to cell division [26]. We posit that the conservation of centromeric sequences between lineages will be reinforced the more reuse the breakpoints associated with these centromere locations experience. This hypothesis can be tested in models where centromere reuse is high, as the evolution of centromeric repeats will be predicted to accompany the evolution of syntenic block rearrangements. The data presented herein was used to determine whether the evolutionary trajectory of centromeric sequence composition has paralleled chromosome evolution, and thus syntenic block arrangement, or whether it follows species evolution.

Previous work on the macropodine species *Macropus rufogriseus *(red-necked wallaby, Mrb) detailed the sequence and karyotypic distribution of three centromeric sequence classes, a functional 178 bp centromere satellite (typified by the sequence Mrb-sat23), a repeat derived from a simple 7-mer (typified by the sequence Mrb-B29), and a degenerate pericentric satellite (typified by the sequence Mrb-sat1)[27]. In the present study, we examined the karyotypic distribution of these three centromeric constituents across *Macropus *and have identified patterns of chromosome distributions. A gene phylogeny based on mitochondrial and nuclear sequences was constructed for nine species within *Macropus*. This phylogeny was then tested for concordance with a phylogeny derived from syntenic block arrangement as determined by GRIMM (Genome Rearrangements In Man and Mouse) algorithms within the Multiple Genome Rearrangements (MGR) program [28]. Comparative analyses of these datasets showed that the evolution of centromeric sequence composition has paralleled chromosome, and thus syntenic block, evolution but has not strictly followed species evolution as measured by phylogenetic metrics.

## Results and discussion

### Sequence analyses

The entire mitochondrial *Cytochrome b *(*Cyt b*) gene was sequenced from eight *Macropus *species (*M. robustus*, *M. antilopinus*, *M. rufus*, *M. giganteus*, *M. eugenii*, *M. rufogriseus*, *M. agilis*, *M. parma*) and *W. bicolor*. One *Petrogale *(*P. xanthopus*) and one *Thylogale *(*T. thetis*) species were sequenced as outgroups (reviewed in [18]), representing two other macropodine genera (Additional data file 1). *Thylogale *carries the ancestral karyotype of all Macropodidae and shares a common ancestor with *Macropus *and *Petrogale *[16,18], rendering it an ideal outgroup taxa for all datasets of our study. The macropodine *Cyt b *is 1,146 bp in length and was included in its entirety for these analyses. The polymorphic sites, 362 variable sites and 200 parsimony informative sites, across the 11 species analyzed in this study were evenly distributed throughout the gene (data not shown).

Due to the potential for natural interspecies hybridization within the *Macropus *genus that may skew mitochrondrial gene sequence towards one species, albeit a very rare occurrence in this clade [29], a nuclear gene, *selenocysteine tRNA *(*TRSP*), and its flanking regions were included in these analyses. *TRSP *is the gene region that includes the *selenocysteine tRNA *gene, an alternative tRNA for the UGA termination codon (also called the opal suppressor) in selenoproteins. Similar in structure to cysteine, selenocysteine substitutes sulfur with selenium. The *TRSP *region was selected because previous studies indicated that regions flanking the *TRSP *transcription unit carried sufficient informative sites for phylogenetic resolution of closely related species within the Canidae [30]. The time since species divergence within the Canidae (0.3-12 million years ago) [31,32] is similar to that of *Macropus *(4-11 million years ago) [18,19]. Moreover, extensive karyotype rearrangement has also been documented across Canid species [33]. Bardeleben *et al*. [30] found the evolutionary rates of the 5' and 3' flanking regions of *TRSP *to be faster than that of introns of some nuclear genes, making the choice of this gene more attractive for our intra-genus study.

**Figure 1:**
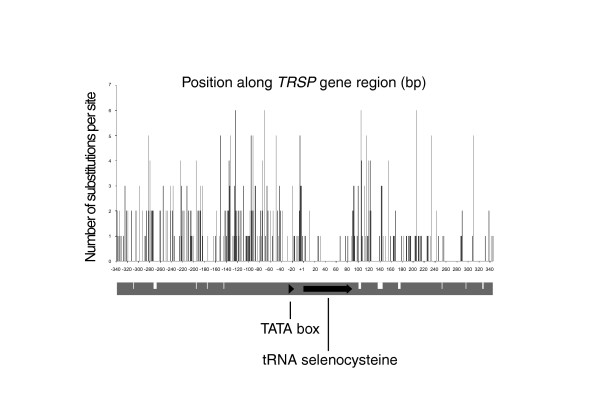
Nucleotide substitutions in *TRSP*. Plot of the number of nucleotide substitutions per site versus position along consensus of *TRSP *across alignment of 11 surveyed species. The tRNA selenocysteine coding region and TATA box are labeled with black arrows. White dashes in the grey gene region represent the position and size of indels, the smallest indel being 1 bp, the largest being 8 bp.

The 87 bp *TRSP *gene and its 5' (340 bp) and 3' (261 bp) flanking regions were sequenced from 11 species (Additional data file 1). Across the dataset, the *TRSP *region (688 bp) had 213 variable sites and 103 parsimony informative sites. Unlike the informative sites identified in *Cyt b *that show an even distribution across the entire gene (data not shown), the informative sites within *TRSP *are clustered in the regions flanking the *TRSP *coding sequence (Figure 1). Nine of the variable sites were intragenic, of which eight were transversions. Five indels were present in the 5' region and six in the 3' region. Within the 5' flanking region, though they do not have good sequence identity, the proximal and distal sequence elements can be found at the same positions as their eutherian counterparts. While the proximal and distal sequence elements are not well conserved, other regions, such as -182 bp to -145 bp upstream, are (Figure 1), but no established functionality has been attributed to them.

### Phylogenetic analyses

**Figure 2:**
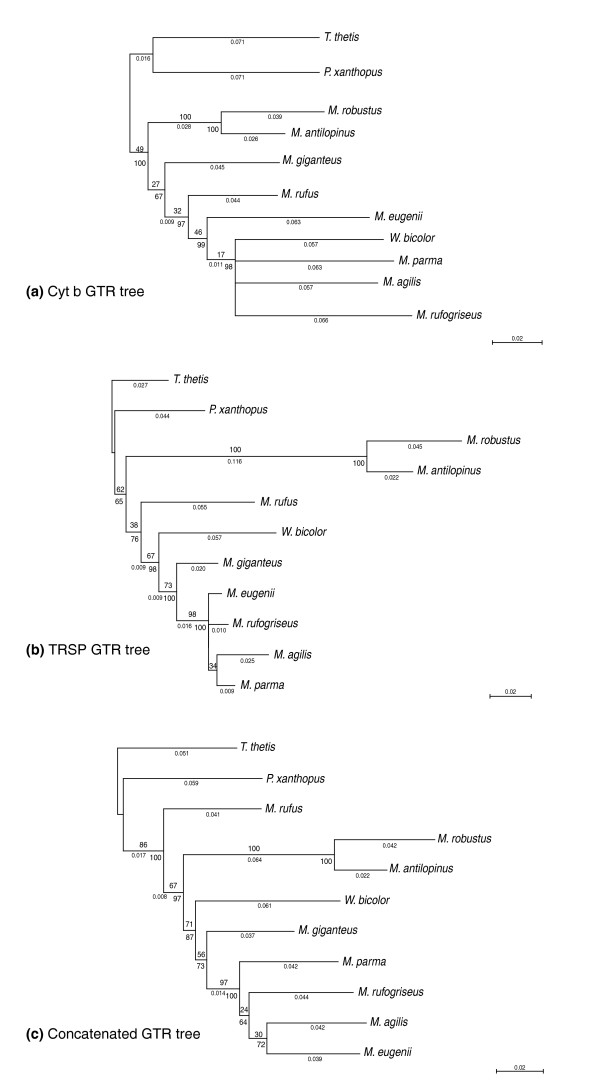
Gene phylogenies. MrBayes GTR/ML phylogenies of *Macropus ***(a) ***Cyt b*, **(b) ***TRSP *and **(c) **concatenated *Cyt b *+ *TRSP*. MrBayes credibility values are listed below the branches, ML bootstrap values above the branches, and branch lengths are shown in italics. The *Cyt b *and *TRSP *trees agree on their placement of *M. robustus *and *M. antilopinus *(the wallaroos) as the sister group, though disagree on their placements of *W. bicolor*, *M. giganteus *and *M. rufus*. The concatenated tree reconciles the two divergent trees, placing *M. rufus *as sister-species to the rest. Distances below 0.008 are not shown, and clade credibility values below 60 are not shown.

Previous studies have failed to clarify the phylogenetic relationships amongst the five groups of *Macropus *species included in this study: grey kangaroo (*M. giganteus*), red kangaroo (*M. rufus*), the wallaroos (*M. antilopinus *and *M. robustus*), swamp wallaby (*W. bicolor*), and the 'true' wallabies (*M. parma*, *M. eugenii*, *M. agilis*, *M. rufogriseus*). The *Cyt b *analysis places *W. bicolor *within the 'true' wallabies, to the exclusion of *M. giganteus*. The p-distances within the *Cyt b *dataset for *W. bicolor *are low and comparable to those of the other wallabies (Figure 2a). The two wallaroo species maintain a close association and define a group unto themselves. In contrast, the *TRSP *tree places *M. giganteus *with the 'true' wallabies, to the exclusion of *W. bicolor *(Figure 2b), with more significant clade credibility values. The tree derived from analysis of a concatenation of both sequences maintains the topology of the *TRSP *tree with respect to these two species and has strong supporting credibility values (Figure 2c). Given the individual or concatenated datasets, we used the Shimodaira-Hasegawa test [34] (as implemented using TREE-PUZZLE 5.2 [35]) to explore the confidence set of phylogenies. Shimodaira-Hasegawa testing of single and combined datasets did not significantly reject phylogenies where *M. giganteus *and *W. bicolor *formed a distinct clade and thus cannot reject the possibility that *M. giganteus *and *W. bicolor *form a separate clade.

**Figure 3:**
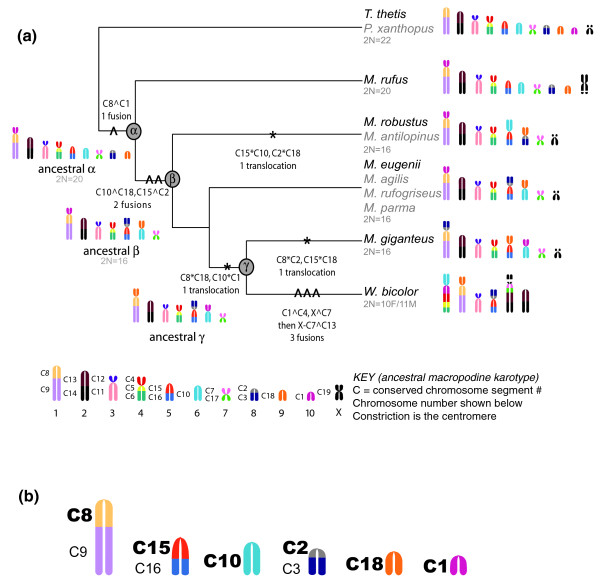
MGR parsimony tree of syntenic block rearrangements. **(a) **Syntenic blocks are colored according to homology for the karyotype of the last common ancestor (represented by *T. thetis*, see KEY). The diploid number is indicated in grey below the karyotype. The number of rearrangements is indicated on branches as fissions (/), fusions (^), and translocations (*). Unmarked branches have no changes. Estimated ancestral karyotypes, constructed by MGR, are included at marked nodes α, β, and γ, without sex chromosome inference. Note that the *M. rufus *karyotype is equivalent to ancestral α, the *M. eugenii *karyotype is equivalent to ancestral β, and the ancestral γ karyotype has no extant counterpart. Nine rearrangements reconstruct all six karyotypes of the genus. The *M. eugenii *autosomal karyotype is equivalent to those of the true wallabies; the *M. robustus *autosomal karyotype is equivalent to the wallaroos herein. Spacers on the X chromosomes represent the positions of the nucleolous orgranizer regions [17]. **(b) **Syntenic blocks active in rearrangements: C1 (dark pink), C2 (grey), C8 (light yellow), C10 (aqua), C15 (dark orange), C18 (orange). The syntenic block numbering is based on the relation to the 2n = 14 ancestral karyotype (see the errata in [11]).

The association supported by the *TRSP *and concatenated datasets places *M. rufus *as sister taxa to all other *Macropus *and *Wallabia*. Surprisingly, in trees produced from both *Cyt b *and *TRSP*, the wallaroos are sister taxa to *Wallabia *and the rest of *Macropus*, though overall sequence differences of *M. rufus *outweigh that association in the concatenated tree (Figure 2a,b versus 2c). The concatenated tree logically places *M. rufus *on the ancestral *Macropus *node, places the two wallaroo species together and places all the 'true' wallabies together (see below and Figure 3). Neither the *Cyt b *nor *TRSP *analysis alone resolves the species within the 'true' wallabies. It is likely that there were not enough phylogenetically informative sites to resolve this group due to their recent derivation, though the combination of both genes does provide some resolution. However, the phylogeny presented is statistically robust (see Materials and methods), and thus provides a sound topological basis from which to examine the pattern of karyotypic evolution in this group.

### Multiple genome rearrangement analysis

Basic marsupial karyotypes can be defined by the arrangement of 19 conserved chromosome segments, or syntenic blocks derived from a common ancestor [11,12] (Figure 3a key). These syntenic blocks can be used to trace the history of chromosome rearrangements across *Macropus *species. The syntenic blocks are arranged in six different configurations among the eleven species examined. Though the karyotypes of most *Macropus *species have been defined by G-banding [13], deriving the most parsimonious chromosome phylogeny has produced conflicting trees [11,13] largely due to a lack of consensus on the amount of convergent breakpoint reuse and the number of translocations across species with a 2n = 16 diploid number. Resolution has been further confounded by the lack of a comprehensive gene phylogeny to use as a guide from which to study the order and pattern of rearrangement of affected chromosomes.

As a general measure of chromosome evolution, a karyotype phylogeny has been generated for *Macropus*. The MGR program [28] was used to reconstruct the most parsimonious lineage for *Macropus *based on syntenic block organization. For this analysis, each chromosomal segment was coded by syntenic number relative to the ancestral karyotype, represented by *T. thetis*, and oriented relative to the centromere position (Figure 3a key). The MGR program allows break-point reuse, fissions, fusions, inversions and translocations to occur in any direction necessary to achieve maximum parsimony [28]. Our analysis of the phylogenetic history of the 19 synteny blocks across this group of mammals supports a tree for the taxa that reduces the number of rearrangements suggested by previous phylogenies [11,13] and supports convergent breakpoint reuse at the centromeres among syntenic blocks C1 (Figure 3a,b, dark pink), C2 (grey), C8 (light yellow), C10 (aqua), C15 (dark orange), and C18 (orange). These six blocks, each with boundaries at the centromere, are involved in the various suites of chromosome rearrangements within this genus.

The MGR analysis of syntenic block rearrangement produced an unrooted tree with three ancestral nodes from which the input karyotypes derive (Figure 3a). Nine steps (one step = one fission, fusion, or translocation) were extrapolated along the total length of the tree. This tree also infers three ancestral karyotypes, denoted as α, β and γ. The ancestral α karyotype, presumed to be the oldest from its proximity to *T. thetis *(an outgroup and a species carrying the 2n = 22 karyotype ancestral to all Macropodidae), is 2n = 20 and equivalent to *M. rufus*. In the ancestral α (and *M. rufus*) karyotype, syntenic block C8 has undergone a fusion with block C1. Ancestral α is inferred to have undergone two autosomal fusions (denoted by '^') to create the 2n = 16 karyotype of ancestral β (C10^C18, C15^C2), equivalent to that of *M. eugenii*. From ancestral β, a further translocation (denoted as '*'; C15*10, C2*C18) achieved a karyotype equivalent to that of *M. robustus*. A different translocation (C8*C18, C10*C1) occurred to form the ancestral γ karyotype, still a 2n = 16 form. The karyotype of *M. giganteus *is derived by yet a different translocation event (C8*C2, C15*C18), still preserving the 2n = 16 form. The karyotype of *W. bicolor *is achieved from the ancestral γ by three fusions, including the fusions of two autosomes to the X.

**Figure 4:**
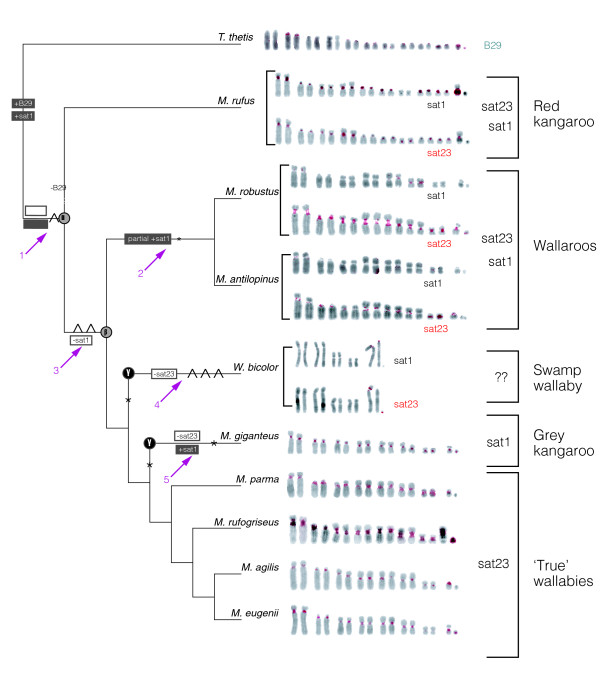
Synthesis of phylogenetic and karyotypic data. Tree topology from Figure 2c overlaid with chromosome rearrangements from Figure 3 and patterns of sequence expansion and contraction as seen from FISH with sat1, B29 and sat23 (right) probes to full karyotypes of representative *Macropus *species. Predominant FISH images are shown on the right, and chromosomes are ordered sequentially (see Additional data files 3-5 for the complete set). Ten rearrangements are indicated on branches as fusions (^) or translocations (*). The positions of the ancestral karyotypes α, β, and γ relevant to this tree are marked. Predominant centromere satellite accretion (+, gain) and diminution (-, loss) is marked by grey and white boxes. The incongruity between the concatenated gene phylogeny and the MGR tree is that the γ ancestor karyotype must have been formed twice and independently in the *W. bicolor *and *M. giganteu*s lineages as shown here. Numbered arrows identify chromosome rearrangements coincident with shifts in satellite content. The major satellite constituent for each macropodine group is indicated to the far right.

**Figure 5:**
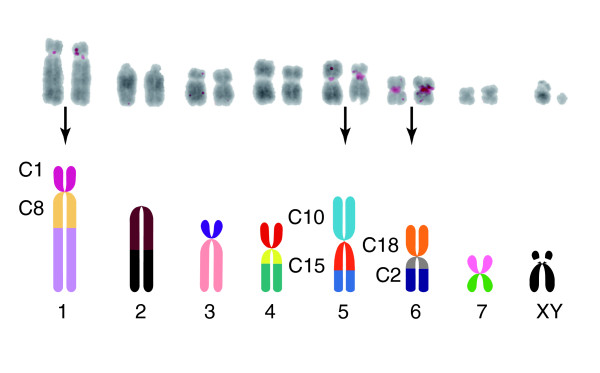
Satellite shifts and breaks of synteny. Satellite shift coincident with breaks of synteny at C8, C15, C10, C2, C18, and C1 in *M. robustus*. Top panel: FISH analysis of sat1. Bottom panel: karyotype of *M. robustus *with the chromosome segments involved in breakpoint reuse indicated in bold.

The MGR derived phylogeny is not concordant with the gene tree (Figure 2c) as it places *M. giganteus *and *W. bicolor *in a separate monophyletic group derived from the γ ancestral karyotype. When the derivation of these arrangements, including the inferred ancestors, is mapped along the tree derived from our sequence analyses, convergent breakpoint reuse is implicated (Figures 3, 4, 5). The breaks between blocks C1, C2, C8, C10, C15 and C18 are used in the derivation of the β ancestral karyotype in the lineage leading to the *M. eugenii *group as well as in the derivation of the γ ancestral karyotype in the lineages leading to *M. giganteus *and *W. bicolor*.

### Fluorescence *in situ *hybridization of centromere satellites

**Figure 6:**
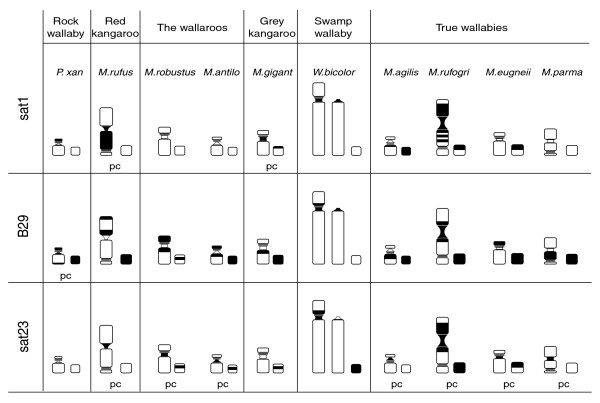
Sex chromosome composite idiogram of probe localization by FISH. Centromeres are represented as small, constricted blocks, and nucleolous orgranizer regions, often found proximal to the centromere, are represented by three thin lines. Chromosomal location of hybridization is indicated in black. Each image was made from observations of ten metaphase spreads. The relative sizing of X chromosomes is according to [17] and this study. Vertical lines separate relative groups of species based on probe distribution in the X and Y. Predominant centromeric probes (pc) indicate hybridization of probe to all autosomal centromeres of the species.

Our MGR analysis, taken in the context of species evolution, indicated that convergent breakpoint reuse has occurred several times within this genus. Moreover, the rearrangements within *Macropus *that distinguish each karyotype involve a breakpoint at a centromere. With the exception of the *W. bicolor *X, which has two autosomes fused to it [36], this karyotypic lability is derived from intrachromosomal rearrangements of six syntenic blocks (C1, C2, C8, C10, C15 and C18) that border active centromeres in all macropodine species (Figure 3b). Each species within *Macropus *also carries a unique X chromosome arrangement and structure (see below and Figure 6), yet is composed of only one syntenic block (C19). For these reasons, we wanted to investigate genetic markers that would prove informative for analyses of sex chromosome evolution in addition to karyotypic evolution of the autosomal complement.

To track the evolution of centromere sequences, with attention towards breakpoint reuse and chromosome rearrangement, as well as sex chromosome structure, we analyzed the chromosome distribution of large blocks of the three centromere satellite classes, sat1, sat23 and B29 [27]. Fluorescence *in situ *hybridization (FISH) of these three satellite classes previously proved informative in the identification of functional centromere sequences within the 2n = 16 *M. rufogriseus *karyotype [27].

A representative of each of these satellite classes was used as a probe for FISH on metaphase chromosome preparations from eight *Macropus *species (*M. eugenii*, *M. agilis*, *M. rufogriseus*, *M. parma*, *M. giganteus*, *M. robustus*, *M. antilopinus*, *M. rufus*), *W. bicolor *and *P. xanthopus *(Figure 4, Additional data files 1-3). The Mrb-sat1 probe is a degenerate pericentric satellite from *M. rufogriseus*, containing just over two 342 bp tandem repeats of 71% homology to one another. The 410 bp Mrb-B29 probe contains simple, tandem 6- and 7-mer repeat variants of GGAATTT. The Mrb-sat23 probed contains one and a half units of a 178 bp alphoid satellite containing a functional CENP-B-box [27].

Figure 4 shows the FISH data for satellites found at all centromeres within a karyotype while Figure 6 summarizes the distribution of all three satellites to the X and Y chromosomes of each species. Within *M. rufogriseus*, *W. bicolor *and *M. giganteus*, sat1 is centromeric on the X chromosomes. *M. giganteus *and *M. rufus *are unique in sharing the localization of sat1 to nearly all autosomal centromeres (Figures 4 and 6). Hybridization of this satellite to *M. rufus *chromosome 7 could not be detected, perhaps because of the small size of the centromere on this chromosome. The ubiquitous presence of sat1 at the centromeres of *M. giganteus *indicates this sequence may be acting as a functional centromere in this species.

The sat1 hybridization to wallaroo *M. robustus *chromosomes is a prominent indicator of the governance rearrangements of the six syntenic blocks (C1, C2, C8, C10, C15 and C18) may have over centromere sequences (Figure 5). The only centromeres to contain sat1 (chromosomes 1, 5 and 6) are the centromeres that separate syntenic blocks C1, C2, C8, C10, C15 and C18. These are the only actively rearranging blocks in the karyotype of this lineage, indicating that the use of these centromeres as sites of rearrangement has led to the conservation of this satellite sequence. *M. antilopinus*, while closely related to *M. robustus*, shows a similar distribution of sat1, although the hybridization signal is more dispersed. Sat23 signal is also reduced in *M. antilopinus*, perhaps an indication of a lesser amount of heterochromatic material. In fact, hybridization signal of centromere satellites in general will be less detectible in *M. antilopinus *as its centromeres in general are smaller than those of *M. robustus*.

Traces of B29, the simple repeat, are seen at the centromeres of *P. xanthopus*, indicating this satellite is not restricted to the *Macropus *(Figure 4). Within the other wallabies (for example *M. eugenii*) this sequence is lost at the X centromere core, but is present pericentromerically (Figure 6, Additional data files 3-5). Remarkably, B29 is conserved as a major component of the Y chromosome in every species included in this study (Figure 6). Its predominance in the *P. xanthopus *and presence in the *Macropus *Y chromosomes implies that once this repeat has been introduced to the Y chromosome, it has not been removed over the evolutionary time period analyzed herein (approximately 15 million years).

Sat23, which contains a CENP-B binding domain in the *M. rufogriseus *variant, does not appear to be present in large enough tandem arrays in *P. xanthopus *to be detected via FISH analyses (Additional data files 3-5), though Southern analyses detect its presence in lower copy number (Additional data file 6). Sat23 is present at every autosomal centromere except for those of *W. bicolor *and *M. giganteus *(Figure 4). However, this satellite is present at the centromere of the X and Y1 of *W. bicolor *and the centromere of the Y of *M. giganteus *(Figures 4 and 6). The presence of sat23 in tandem arrays exclusively at all centromeres of most *Macropus *species (Figure 4, Additional data files 3-5) indicates that this genus is characterized by a conserved centromeric sequence. It is important to note that given the low stringency applied to our FISH assays, these sequences are likely not identical but are homologous and only detectable in large repeated blocks.

The pattern of satellite expansion and contraction, taken in the context of tree topology and chromosome rearrangement (Figure 4) indicates that the amounts of both sat1 and sat23 have each grown and diminished in divergent lineages that have experienced different types of chromosome rearrangements. For example, the contraction of sat23 in *W. bicolor *and *M. giganteus *coincides with the formation of the ancestral γ karyotype through the same translocation of (C8*C18) and (C10*C1) (Figure 3). Subsequent to this, sat1 experienced another expansion specific to the *M. giganteus *lineage and coincident with one more translocation between (C8*C2) and (C15*C18) (Figure 3). Prior to the divergence of *M. rufus *an expansion of sat23 and the reduction of B29 coincides with a fusion (C1^C8). The wallaroos (*M. robustus *and *M. antilopinus*) experience a partial increase of sat1 accompanying a translocation (C15*C10, C2*C18) in their karyotype lineage only in the centromeres participant in common translocation sites (the centromeres between C1 and C8, C15 and C10, and C2 and C18) (Figures 3, 4, 5).

Every change in karyotypic evolution in this genus has been accompanied by a corresponding change in predominant centromeric sequence composition. From our data it appears that the contractions of centromere satellites are most often associated with fusion events during chromosome rearrangement, and the expansion of centromere satellites are most often associated with translocation events. The amount of satellite B29 diminishment coincides with a fusion at arrow 1 in Figure 4. Reduction of satellites sat1 and sat23 coincide with fusions at arrows 3 and 4, respectively. Arrows 2 and 5 indicate sat1 accretion accompanies translocation events. On the lineage to *W. bicolor *(arrow 4) a translocation also occurs. While we have been able to identify a centromeric repeat that has undergone diminution associated with the fusion, we have not been able to identify a predominant centric sequence nor its accompanying accretion in this lineage as predicted by the presence of the translocation. It is likely that an as yet unidentified sequence exists, and we predict it has undergone an expansion to become more prevalent at the centromeres of this species.

## Conclusion

### Phylogenetic history of *Macropus*

While several studies have attempted to refine a phylogeny for the genus *Macropus*, these studies were lacking in either species coverage or support. Previous morphological and serological studies of *Macropus *adequately sampled the genus, though were lacking in statistical support [22,23,37]. Previous genetic studies, while statistically supported, lacked adequate representation of the genus [18]. The choice of two genes, one mitochrondrial and one nuclear, provides for a sound phylogenetic analysis of this group of species [38-58]. The *Cyt b *and *TRSP *gene phylogenies reported herein include 9 of the 13 extant species (approximately 70% coverage), encompassing a more comprehensive dataset for developing a *Macropus *phylogeny. Our phylogenetic analyses derived from the *Cyt b*/*TRSP *concatenated dataset shows high Bayesian clade credibility values and maximum likelihood (ML) boostrap values (Figure 2c), providing a robust phylogeny on which to analyze the pattern of centromere and chromosome evolution across this group of mammals.

The placement of *W. bicolor *in relation to the *Macropus *phylogeny has previously been debated [19]. *W. bicolor *is the only extant member of its genus. The status of this species as a sister genus to *Macropus *has been historically supported by morphological [19], immunological [22], and limited gene phylogeny studies [18]. However, this placement has been challenged by serology [23] and DNA-DNA hybridization [19,20,37], providing support for inclusion of *Wallabia *species within *Macropus*. Our gene based phylogenies (Figure 2) and MGR karyotype analysis (Figure 3) find *Macropus *to be monophyletic including *Wallabia*.

Another conclusion from these phylogenetic analyses is the relative position of *M. rufus *(red kangaroo) and *M. giganteus *(grey kangaroo). The *Cyt b*/*TRSP *tree (Figure 2c) excludes *M. rufus *from the rest of *Macropus*, while placing *M. giganteus *and *W. bicolor *with the 'true' wallabies. Previous taxonomists placed *M. rufus *with the wallaroos in a separate genus, *Osphranter *[19]. The analyses presented herein do not place these three species into one monophyletic group and support their inclusion within *Macropus*.

### Chromosomal history of *Macropus*

Comparison of the *Cyt b*/*TRSP *phylogeny (Figure 2c) overlaid with the karyotype analysis derived from the MGR phylogeny (Figure 3) indicates ten different rearrangements are needed to form every karyotype derived from the ancestral α karyotype (Figure 4). Though the representation of chromosome rearrangements with respect to the gene phylogeny is less parsimonious than the MGR analysis by one step, it is probably more reflective of the natural history of the clade as measured by the *Cyt b*/*TRSP *analysis and FISH analyses.

In our analyses we examined the evolution of centromere satellite repeats across *Macropus *species to determine whether the path of centromere evolution has paralleled chromosome evolution or species evolution, as measured by gene histories. The distribution of predominant centromeric sequences across these species is not informative when mapped onto the gene phylogeny alone (Figure 4). When the history of syntenic block rearrangement is considered, the contractions and expansions of predominant satellites are found to consistently accompany specific karyotype rearrangements of syntenic blocks C1, C2, C8, C10, C15 and C18 (see Figure 5 for an example). Thus, there is a strong correlation between changes in predominant satellite sequences, with respect to homogenous distribution across all centromeres within a karyotype, and chromosome rearrangement events.

Of significance is the demonstration that convergent breakpoint reuse between C1, C2, C8, C10, C15 and C18 results in convergent centromere restructuring. Other studies have identified retention of low-copy numbers of sat23 satellite sequence at the breaks of synteny between most of the 19 conserved chromosome segments within *M. eugenii *[6]. Based on evidence of convergent centromere sequence expansion of sat1 among *M. rufus*, *M. robustus*, and *M. giganteus *(Figure 4), we hypothesize that retention of these sequences at breaks of synteny in low copy provides the sequence targets for centromere satellite expansion.

Our data suggest that 'new' satellite sequences have not been repeatedly introduced into the macropodine genome to become predominant centromeric sequences as predicted by centromere drive [59]. Rather, these centromeric satellites remain in the genome, likely at latent centromere locations [6], and undergo recurrent repeat copy number expansion and contraction in divergent lineages. This analysis does not imply *de novo *adoption of previously non-centromeric sequences at centromere locations following chromosome rearrangement, but indicates the same sequences can undergo convergent expansion across all centromeres in different lineages.

Salser *et al*. [24] proposed the 'library hypothesis' of satellite evolution in which related lineages share a collection of heterochromatic repeat sequences that may become preferentially amplified in any of the given species during the normal events of centromere evolution. In *Macropus *the 'library' of satellite sequences, including sat1, sat23 and B29, is involved in the creation of large, satellite arrays. This conservation implies that centromeric sequences are not created *de novo*, but recycled from the existing library. Mestrovic *et al*. [60] found support for the 'library hypothesis' in examining satellite sequence predominance across congeneric species of *Palorus *insects. By PCR assays it was determined that though all *Palorus *species examined possessed all satellites examined, a different single satellite was greatly amplified in each of the different species, demonstrating that all species shared a common satellite library from which the amplifications occurred. We have found the same to be true by FISH (Figures 4 and 5, Additional data files 3-5) and Southern analyses of our repeats across *Macropus *(Additional data file 6). Lin and Li [61] identified similar inter-genera evidence of centromeric heterochromatin conservation among cervid deer.

Within *Macropus*, the recurrent pattern of detectable repeat presence or absence by FISH in the autosomes versus the sex chromosomes (Figures 4 and 6) could be indicative of the rate at which these two types of chromosomes accumulate and dissipate centromeric material. The sex chromosomes of this group appear to retain tandem arrays of ancestral centromeric material for longer periods of time. For example, the presence of B29 on the sex chromosomes of all species examined indicates an origin for this satellite predating *Macropus *diversification. While most *Macropus *species carry all three satellites on their sex chromosomes, subsequent reduction of sat1 on the sex chromosomes has occurred in the lineage leading to *M. robustus/M. antilopinus *as well as within *M. parma*. Most species within *Macropus *carry a suite of satellites (B29, sat1) on their sex chromosomes that are no longer found as expanded satellites on their autosomal counterparts. Evidence from cervid deer and muntjac also shows retention of tandem arrays of satellites in the sex chromosomes that are not found in the autosomes [61,62].

Mechanistic processes inherent to fusion and translocation events may be responsible for the observed contractions and expansions of the satellite arrays. Diminution of satellite arrays by excision may occur as a result of subtractive processes occurring during fusion events. Prior to Robertsonian fusions, chromosome breaks within the centric satellites remove the p-arms, and a portion of the centromere and pericentromere, to expose the fusion sites [63,64]. The centric position of the break sites leads to overall reduction of satellite sequences as a result of fusion events.

In contrast, duplication of centromere material leading to accretion of satellite arrays may be the result of arm-swapping translocations. Studies of patterns of segmental duplications in humans indicate that segmental duplications precede and accompany translocation events [8]. Segmental duplications show a concentration near centromeres and occur more often interchromosomally. As such, they are hypothesized to aid in centromere sequence convergence [9,65,66]. Segmental duplications preceding a translocation event would increase sequence identity between sites, making such translocations events more likely [67]. At the centromeres, such duplication events would also serve to distribute satellite sequences to centromeres throughout the genome, increasing the likelihood of the adoption of a predominant satellite sequence [2]. Propagation of satellites via segmental duplication events also supports the 'library hypothesis' of centromere satellite origination, as it represents a recycling process inherent to the hypothesis.

After predominant centromere satellites were identified in a majority of the genus, these sequences were used to track centromere evolution. Comparing the karyotype phylogeny to the gene-tree topology concludes that while *Macropus *species possess several divergent karyotypes, there is reuse of satellite sequences as a result of breakpoint reuse at specific syntenic block boundaries coincident with centromeres. This study shows that the 'library hypothesis' describes the patterns of centromere sequence convergence within this mammalian lineage. Thus, satellite sequence evolution is found to strictly follow chromosomal evolution, likely as a result of the dynamic role the centromere plays in karyotype change.

## Materials and methods

### *Cyt B *sequence analyses

Mitochondrial *Cyt b *was amplified from 11 species (Additional data file 1) using the primers Mr1/Mr2 [68] (Additional data file 7) that flank the gene. Internal gene sequencing was done with combinations of primers (Additional data file 7). Products were direct sequenced with ABI BigDye3.1 on an ABI 3130 sequencer as per the manufacturer's instructions (ABI: Foster City, CA, USA). *Cyt b *was sequenced from two individuals per species to confirm species identity. However, two individuals each were unavailable for *M. antilopinus *and *M. parma*. Thus, branch lengths based on the number of substitutions per site were calculated to evaluate intra-versus interspecies diversity across the first 406 bp of *Cyt b *from all available Macropodine sequences available from GenBank and our dataset (Additional data file 1). All interspecies branch lengths were larger than all intraspecies branch lengths. The distances between the respective nearest neighbors to *M. antilopinus *and *M. parma *exceeded all intraspecies branch length values and thus were concluded to be appropriately individual species, at least sharing identity with none of the species included in this study. *P. xanthopus *and *T. thetis *were sequenced as outgroups.

### *TRSP *sequence analyses

*Mus musculus *(mouse), *Oryctolagus cuniculus *(rabbit) and *Gallus gallus *(chicken) sequences of the 87 bp *TRSP *transcription unit were used to search the NCBI Trace Archives of *M. eugenii*. The Trace Archives' sequences of *M. eugenii *with the highest percent identity to the mouse, rabbit and chicken *TRSP *were aligned with VectorNTI version 10 (Invitrogen: Carlsbad, CA, USA) to find the largest contiguous trace sequence containing *TRSP*.

The *M. eugenii *trace sequence accession number [Genbank:976005645] was found to have the highest percent identity to both the other trace sequences from *M. eugenii *as well as *TRSP *from mouse and rabbit. Primer3 [69] was then used to construct primers from [Genbank:976005645] spanning the *TRSP *gene region (Additional data file 7). Primers to the *TRSP *gene itself were designed using the *Ornithorhynchus anatinus *(platypus) trace sequence [Genbank:188164072] (Additional data file 7).

The nuclear gene region of *TRSP *(688 bp) was sequenced from two individuals of each species. Direct sequencing was performed as above. Within *W. bicolor*, *M. robustus *and *M. antilopinus*, some regions of this gene were not amenable to direct sequencing and, thus, were subcloned into pGEM-T Easy (Promega: Madison, WI, USA) and then sequenced in triplicate from the plasmid clones. The *TRSP *gene from *P. xanthopus *and *T. thetis *were PCR amplified and direct sequenced as outgroups.

Alignments were performed in Clustal X [70]. MEGA 3.1 was used to find the number of informative sites and proportional distance values for both genes [71] (Figure1).

### Phylogenetic analyses

Identical tree topologies were generated using MrBayes [72,73], Mega [71] and PhyML [74]. Analyses performed were used to infer phylogenetic relationships for *Cyt b*, *TRSP*, and *Cyt b-TRSP *concatenated together. *T. thetis *and *P. xanthopus *are outgroups to the *Macropus *dataset. Within MrBayes, the general time reversible (GTR) model gave the greatest ML and clade credibility values across all three datasets. Five Markov Chain Monte Carlo chains were run for 1,000,000 generations, sampling every generation with a burn-in of 1,000 generations. Potential scale reduction factor values all converged on 1.000 by the conclusion of the runs. For each of the *Cyt b *and *TRSP *datasets, tree topology did not change with respect to model choice. When the two datasets were concatenated to form a contiguous sequence, only the supported tree topology within the wallabies changed with respect to the model.

ML trees were generated in PhyML [74] using the following parameters: GTR nucleotides substitution model, discrete gamma model, 4-categories, shape parameter, proportion of invariant sites and nucleotide frequencies estimated from the data. Bootstrap values were also generated in PhyML (shown in Figure 2). The ML trees, and an additional 18 tree topologies obtained through rearrangement, were used for the Shimodaira-Hasegawa test [34] in TREE-PUZZLE 5.2 [35].

### Multiple genome rearrangements tool

The web-based software MGR [28,75], designed for constructing phylogenies based on gene order for multichromosomal rearrangements, was used to construct a phylogeny based on syntenic block rearrangement across the clade (Figure 3). Only fissions, fusions, inversion and translocations are considered significant. Rearrangement events between syntenic blocks or chromosomes occur one at a time. As per the rules of this program, no unitary associations were locked, meaning breakpoint reuse was allowed. The input was oriented relative to the arrangement of the *Thylogale *syntenic arrangement and the output was an unrooted parsimony tree.

*Thylogale*, a macropodeid possessing the ancestral familial karyotype and diploid number of 2n = 22, was coded such that each syntenic block was oriented and numbered according to [11]. *T. thetis *also shares its autosomal karyotype with *P. xanthopus*. All other species included in this analysis experienced reductions in chromosome number relative to *T. thetis *and were coded relative to this ancestral form. The 2n = 16 karyotype of *M. eugenii *is shared with *M. agilis*, *M. rufogriseus *and *M. parma*. The 2n = 16 karyotype of *M. robustus*, defined by a different suite of fusions, is shared with the other wallaroo species, *M. antilopinus*.

### Cross species fluorescence *in situ *hybridization

Mrb-sat1, Mrb-B29, and Mrb-sat23 clones, isolated following microdissection of the *M. rufogriseus *X chromosome [27], were PCR labeled with biotin-16-dUTP (Roche: Basel, Switzerland) as per the manufacture's instructions. *M. rufogriseus *FISH were performed as per [27]. *T. thetis *chromosomes were unattainable for this study, though this species has a karyotype configuration equivalent to *P. xanthopus*. All cross-species FISH experiments were hybridized at 37°C in 50% hybridization solution (50% formamide, 2 × SSC, 500 ng/ml salmon sperm DNA, 200 ng probe) and washed at room temperature (three 50% formamide/2 × SSC washes for 5 minutes each, followed by three 2 × SSC rinses at room temperature). Slides were blocked with 4 × SSC/0.2% Tween-20/5% bovine serum albumin before avidin rhodamine (Texas Red; Invitrogen: Carlsbad, CA, USA) incubation at 37°C for 30 minutes. Antibody layering, when needed, was of first, avidin TexasRed, second, anti-rhodamine biotin, and third, avidin TexasRed.

For each species, we determined the most stringent conditions required to obtain FISH signal without losing signal integrity. Hybridization time and layering varied as follows: *M. eugenii *with all three probes, and *M. agilis *with Mrb-B29 and Mrb-sat23 were hybridized for two nights and detected with one layer; *M. parma*, *M. rufus*, and *M. giganteus *with all three probes, *W. bicolor *with Mrb-B29, *M. robustus *with Mrb-B29 and Mrb-sat23, and *M. antilopinus *with Mrb-B29 were hybridized for three nights and detected with one layer; *W. bicolor *with Mrb-sat1 and Mrb-sat23, *P. xanthopus *with Mrb-B29 and Mrb-sat23, and *M. antilopinus *with Mrb-sat23 were hybridized for three nights and detected with three layers.

Because of low signal strength from the sat1 probes, due to cross species variation, sat1 FISH to *M. agilis*, *M. antilopinus*, *M. robustus *and *P. xanthopus *used pooled sat1 probes derived from *M. robustus*, *M. parma*, *W. bicolor*, and *M. rufus *species (probes named Mrob-sat1, Mpm-sat1, Wbi-sat1 and Mrfs-sat1, respectively). The pooled sat1 probes from these species were PCR amplified with Mrb-sat1 primers (Additional data file 7). PCR products were cloned and sequenced, with a range of 74.8-91.5% identity to Mrb-sat1, verifying PCR product identity given the average sequence identity observed for satellites both within one genome (50-100% between different monomers) and between species [76,77]. Clones were PCR labeled for FISH as above. Probes were pooled during precipitation (200 ng of each) prior to rehydration in the hybridization solution. Pooled probes were hybridized for four nights, and detected with three layers, as above. All FISH conditions are described in Additional data file 2.

Slides were mounted with DAPI/Vectasheld (Vector Laboratories: Burlingame, CA, USA) mounting media. Images were captured with a Leica DM6000B microscope with a DFC350FX-R2 digital camera and analyzed with Leica CW4000 Cytogenetics Karyotype software (Leica Microsystems: Bannockburn, IL, USA).

## Additional data files

The following additional data are available with the online version of this paper. Additional data file 1 is a list of all species names and corresponding accession numbers used in phylogenetic studies. Additional data file 2 lists the cross-species FISH hybridization conditions. For each species (left), probes used are indicated (top). For the pooled probe set, a combination of sat1 sequences from Mrob, Mpm, Wbi, Mrfs were used in one hybridization reaction. Hybridization time is indicated by the number (hyb #) of days probe is incubated at 37°C. The number of antibody detection layers is also indicated. All other conditions are described in the Materials and methods. Additional data files 3, 4 and 5 are the FISH for each satellite (sat1, B29 and sat23, respectively) to each species used in these analyses. Probe images are in red and metaphase chromosomes are inverted DAPI. Additional data file 6 shows the Southern analyses of each satellite (sat1, B29 and sat23) to each species used in these analyses. Additional data file 7 is a list of all primer sequences used in sequence and phylogenetic analyses. *Cyt b *nucleotide positions are numbered according to *M. robustus *numbering [GenBank:Y10524]; *Cyt b *spans 14,184 bp to 15,329 bp.

## Abbreviations

*Cyt b = cytochrome b*; FISH = fluorescence *in situ *hybridization; GTR = general time reversible; MGR = multiple genome rearrangement; ML = maximum likelihood; Mrb = *Macropus rufogriseus*; *TRSP = selenocysteine tRNA*.

## Authors' contributions

KVB performed all experiments and analyses herein and wrote the manuscript, GCF performed Southern analyses, MDBE provided tissue and DNA samples and edited the manuscript, and RJO provided general project oversight and co-wrote the manuscript.

## Supplementary Material

Additional data file 1 All species names and corresponding accession numbers used in phylogenetic studies Click here for file

Additional data file 2 For each species (left), probes used are indicated (top). For the pooled probe set, a combination of sat1 sequences from Mrob, Mpm, Wbi, Mrfs were used in one hybridization reaction. Hybridization time is indicated by the number (hyb #) of days probe is incubated at 37°C. The number of antibody detection layers is also indicated. All other conditions are described in the Materials and methods. Click here for file

Additional data file 3 Probe images are in red and metaphase chromosomes are inverted DAPI Click here for file

Additional data file 4 Probe images are in red and metaphase chromosomes are inverted DAPI Click here for file

Additional data file 5 Probe images are in red and metaphase chromosomes are inverted DAPI Click here for file

Additional data file 6 Southern analyses of each satellite (sat1, B29 and sat23) to each species used in these analyses Click here for file

Additional data file 7 *Cyt b *nucleotide positions are numbered according to *M. robustus *numbering [GenBank:Y10524]; *Cyt b *spans 14,184 bp to 15,329 bp Click here for file
