# Supplementary material for: Species-specific shifts in centromere sequence composition are coincident with breakpoint reuse in karyotypically divergent lineages
Source: Genome Biol. 2007 Aug 20;8(8):R170. doi: 10.1186/gb-2007-8-8-r170 (PMC2375000; doi:10.1186/gb-2007-8-8-r170)
Supplement: Additional data file 1 — All species names and corresponding accession numbers used in phylogenetic studies [file gb-2007-8-8-r170-S1.doc]

**Additional Data File 1.** Species names and corresponding accession numbers used in phylogenetic studies.

| **Subfamily Macropodinae** | **Group and Common Name** | **GenBank Accession #'s** | |
| --- | --- | --- | --- |
|  |  | **CYTB** | **TRSP** |
| **Genus *Macropus*** |  |  |  |
|  | **WALLABIES** |  |  |
| *M. eugenii* | Tammar Wallaby | EF368028 | EF368049, EF368050 |
| *M. agile* | Agile Wallaby | EF368029 | EF368051, EF368052 |
| *M. parma* | Parma Wallaby | EF368030 | EF368047 |
| *M. rufogriseus banksianus* | Red-necked Wallaby | EF368027 | EF368048 |
|  | **RED KANGAROO** |  |  |
| *M. rufus* | Red Kangaroo | U87136 | EF368041 |
|  | **GREY KANGAROO** |  |  |
| *M. giganteus* | Eastern Grey Kangaroo | EF368023 | EF368045, EF368046 |
|  | **WALLAROOS** |  |  |
| *M. robustus* | Common Wallaroo | EF368025 | EF368043, EF368044 |
| *M. antilopinus* | Antilopine Wallaroo | EF368024 | EF368042 |
|  |  |  |  |
| ***Genus Wallabia*** | **SWAMP WALLABY** |  |  |
| *W. bicolor* | Swamp Wallaby | EF368031 | EF368039, EF368040 |
|  |  |  |  |
| ***Genus Petrogale*** | **ROCK-WALLABIES** |  |  |
| *P. xanthopus* | Yellow-footed Rock-wallaby | EF368032 | EF368037, EF368038 |
| *P. mareeba* | Mareeba Rock-wallaby | EF368033 | - |
|  |  |  |  |
| ***Genus Thylogale*** | **PADEMELONS** |  |  |
| *T. thetis* | Red-necked Pademelon | EF368034 | EF368035, EF368036 |
