# Supplementary material for: Species-specific shifts in centromere sequence composition are coincident with breakpoint reuse in karyotypically divergent lineages
Source: Genome Biol. 2007 Aug 20;8(8):R170. doi: 10.1186/gb-2007-8-8-r170 (PMC2375000; doi:10.1186/gb-2007-8-8-r170)
Supplement: Additional data file 2 — For each species (left), probes used are indicated (top). For the pooled probe set, a combination of sat1 sequences from Mrob, Mpm, Wbi, Mrfs were used in one hybridization reaction. Hybridization time is indicated by the number (hyb #) of days probe is incubated at 37°C. The number of antibody detection layers is also indicated. All other conditions are described in the Materials and methods. [file gb-2007-8-8-r170-S2.doc]

**Additional Data File 2.**

|  | **Probes** | | | |
| --- | --- | --- | --- | --- |
| **Species** | **Mrb-sat1** | **Mrb-B29** | **Mrb-sat23** | **Pooled sat 1***  Mrob-sat1  Mpm-sat1  Wbi-sat1  Mrfs-sat1 |
| *P. xanthopus* | -- | Hyb 3 days  3 layer detection | Hyb 3 days  3 layer detection | Hyb 4 days  3 layer detection |
| *M. rufus* | Hyb 3 days  1 layer detection | Hyb 3 days  1 layer detection | Hyb 3 days  1 layer detection | -- |
| *M. robustus* | -- | Hyb 3 days  1 layer detection | Hyb 3 days  1 layer detection | Hyb 4 days  3 layer detection |
| *M. antilopinus* | -- | Hyb 3 days  1 layer detection | Hyb 3 days  3 layer detection | Hyb 4 days  3 layer detection |
| *M. agilis* | Hyb 3 days  1 layer detection | Hyb 3 days  1 layer detection | Hyb 3 days  1 layer detection | -- |
| *W. bicolor* | Hyb 3 days  3 layer detection | Hyb 3 days  1 layer detection | Hyb 3 days  3 layer detection | -- |
| *M. agilis* | -- | Hyb 2 days  1 layer detection | Hyb 2 days  1 layer detection | Hyb 4 days  3 layer detection |
| *M. rufogriseus* | Hyb 1 day  1 layer detection | Hyb 1 day  1 layer detection | Hyb 1 day  1 layer detection | -- |
| *M. eugenii* | Hyb 2 days  1 layer detection | Hyb 2 days  1 layer detection | Hyb 2 days  1 layer detection | -- |
| *M. parma* | Hyb 3 days  1 layer detection | Hyb 3 days  1 layer detection | Hyb 3 days  1 layer detection | -- |

* Mrob-*M. robustus*; Mpm-*M. parma*; Wbi-*Wallabia bicolor*; Mrfs-*M. rufus*
