# Supplementary material for: Species-specific shifts in centromere sequence composition are coincident with breakpoint reuse in karyotypically divergent lineages
Source: Genome Biol. 2007 Aug 20;8(8):R170. doi: 10.1186/gb-2007-8-8-r170 (PMC2375000; doi:10.1186/gb-2007-8-8-r170)
Supplement: Additional data file 7 — Cyt b nucleotide positions are numbered according to M. robustus numbering [GenBank:Y10524]; Cyt b spans 14,184 bp to 15,329 bp [file gb-2007-8-8-r170-S7.doc]

**Additional Data File 7.** Primer sequences used in sequence and phylogenetic analyses. *Cyt b* nucleotide positions number according to *M. robustus* numbering [GenBank: Y10524], *Cytb* spans 14184bp to 15329bp.

| **Primer** | **5'/3'** | **Sequence 5'-3'** | | **Position** |
| --- | --- | --- | --- | --- |
| **CYTB** |  |  |  |  |
| **External** |  |  |  |  |
| Mr1 | 5' | CATTTTAGTATGGACTCTAACCATAACC | | 14195 |
| Mr2 | 3' | AGGGTGTTATACCTTCATTTTTGG | | 15472 |
| **Internal** |  |  |  |  |
| Mpod cytb int_L | 5' | ACAAAGCCACCCTCACACG | | 14695 |
| Mpod cytb int_R | 3' | GAGTATAAGTATGAGGCCAAGTGC | | 14891 |
| Mpod648_L | 3' | TCTGAGTCGGGGTTGATTC | | 14913 |
| Mpod948R | 3' | ATTAGGCTTCGTTGTTTAGA | | 15130 |
| Mpod cytb intR2 | 3' | GGAGAGAAATTGTCTGGGTC | | 14937 |
| Mpod cytb revout2 | 5' | GACCCAGACAATTTCTCTCC | | 14917 |
|  | | |  |  |
| **TRSP** |  |  |  |  |
| **External** |  |  |  |  |
| MeuTRSP_4L | 5' | ACTCCTCGGGGTCATTCC | |  |
| MeuTRSP_789R | 3' | GATCTCAAAGTATCCGTGAGAGG | |  |
| **Internal** |  |  |  |  |
| TRSPcore_L | 5' | GATGAGCCTCAGTGGTCTGG | |  |
| TRSPcore_R | 3' | GGAATTGAACCACTCTGTCG | |  |
|  |  |  |  |  |
| **Mrb-sat1** |  |  |  |  |
| sat B15 L | 5' | TGCAGTTAGGTAGGCGATATTT | |  |
| sat B15 R | 3' | CATTGCTGCAGATAAGATATGAGG | |  |
